# The System of Nursing at the London Hospital

**Published:** 1887-01-01

**Authors:** Eva C. E. Luckes

**Affiliations:** Matron


### A Noble Profession: Nursing the Sick.

### THE SYSTEM OF NURSING AT THE LONDON HOSPITAL.

By Miss Eva C. E. Lückes, Matron.

**Introductory.** It may be of interest to readers of The Hospital to have a brief sketch of the general system of nursing adopted at the London Hospital, more especially with regard to the training of probationers. The existing regulations for the reception of "regular probationers" were put into practice in November, 1880, so that the first certificates of training gained on the present system were awarded in that month, 1882. From that time up to the present, exactly 150 certificates of varying degrees of merit have been won, the proportion of these increasing the last two years. In all probability, we may reckon that for the future fifty fully trained nurses will receive a certificate of training annually from this Hospital. It should be clearly understood that no certificate is awarded, in any circumstances, under the full term of two years' training, so that, roughly speaking, we may be said to have on our list of "regular probationers," fifty in their first year, and fifty completing their second; the latter, if they have made an intelligent use of their first year's training, being well qualified to act as special nurses on critical cases, or to take "staff-nurse's duty," as required. As the training of each nurse extends over this period of two years, a little careful management enables every individual probationer to obtain an insight into medical and surgical nursing in men's and women's wards, both on day and night duty. A register is kept wherein the name of each probationer, and an exact account of where she has worked from the day of her appointment, are duly recorded. After the month's trial, and subject to the sanction of the physician, who kindly undertakes to judge of the physical fitness of the approved candidates for the work, they are required to sign an agreement to serve the Hospital for two years. They are paid £12 the first year, and £20 the second. The Hospital provides a certain amount of uniform, and finds everything except washing. Every member of the nursing staff is now provided with a separate bedroom, and all the domestic arrangements are exceedingly comfortable.

**Is Two Years enough?** It is open to question whether probationers who receive such advantages should not be bound to serve the Hospital for a longer period than two years. Doubtless in some cases it is disappointing to lose promising workers just when they become qualified to be really of use; but there is, I hope, a growing feeling amongst the best workers, that it is only fair, and indeed a mutual benefit, to remain at the Hospital in which they have been trained, in whatever capacity may be open to them, for the additional year for which they would be actually bound to serve at nearly every other large training school. On the other hand, while we on our part rarely break through the two years' engagement, except for a grave fault or very marked incapacity for the work, we do not feel in the least bound to retain the services of any but satisfactory nurses on our permanent staff. So, taking it altogether, we are inclined to persevere in faith with our present liberal terms, and to continue to add to the other advantages we offer, the fact that our probationers are actually bound to us for two years only.

**The System Explained.** The theoretical portion of a nurse's training is adequately provided for by continuous courses of Lectures, which are given every Wednesday at 8 p.m. Ladies not connected with the Hospital are admitted to these on payment of 10*s.* 6*d.* for each course. The first set of lectures, on General Nursing, is given by the Matron, and begins at the end of August. The second course, on Elementary Anatomy and Surgical Nursing, begins in November, and is kindly given by Frederick Treves, Esq., F.R.C.S., Surgeon to the London Hospital. The third course, on Elementary Physiology and Medical Nursing, beginning in March, is kindly given by A. Ernest Sansom, Esq., M.D., F.R.C.P., Physician to the London Hospital. At the end of June, when the third course of lectures is complete, an examination is held and prizes are given. We cannot speak too gratefully of the kindness of these gentlemen in patiently giving their valuable instructions to fresh sets of probationers year after year; and it is obvious that much of the success achieved by our nurses is due to the unfailing help and active interest displayed by these lecturers, and many other members of the medical staff, in all that concerns the welfare of the nurses or their work. In addition to the weekly lecture, all those whose turn it will be to go in for the ensuing examination are required to attend *small* classes once a week, that they may get the benefit of individual instruction on the subject of the last lecture. Bandaging classes are also held regularly, so that *all* get the opportunity of perfecting themselves in the knowledge they come here to acquire.

**Special Advantages of "The London."** It can scarcely be necessary to refer to the unrivalled advantages for practical experience to be gained in the largest hospital in the kingdom. The busy wards, filled with constantly changing patients, drawn chiefly from the immediate densely populated neighbourhood, afford almost every variety of medical and surgical nursing in a comparatively short space of time. In addition to the wards there is the receiving-room, and the immense out-patient department, to be looked at, from a "nurse's," if not exactly from a nursing point of view. The amount of attendance required in the out-patient department is, of course, very limited as compared with that required in the wards. Nevertheless, the experience to be gained there is complete of its kind, and simply invaluable to all who are training with a special view to parish or district nursing.

**Worthy of General Adoption.** If I may digress for a moment from the subject of the actual training of probationers, I should like to mention that in January, 1884, it was considered desirable to make sundry alterations in the nursing arrangements connected with the receiving-room and out-patient departments of this hospital. A fully-trained sister was appointed to divide her time between the receiving-room and out-patient department—meeting, as far as possible, in the way of superintendence the requirements of each. Every physician or surgeon attending the out-patients has a nurse or probationer waiting upon him and his patients for the allotted time, while the sister supervises the nurses and gives her attention and assistance where found necessary. When this department is closed, these nurses are on duty as extra hands in the wards, so that they are enabled to combine a useful experience of the sick poor as they are to be found in their own homes, with a certain amount of the ordinary hospital experience. The medical staff expressed their cordial appreciation of this arrangement directly it was instituted, observing that having a nurse in attendance was instrumental in saving the doctor's time and in adding to the comfort of the out-patients. I mention this, rather with the object of asking the question—Is this arrangement as universal in hospitals as it might be? The advantage of these opportunities to nurses desirous of qualifying themselves for that branch of their profession known as district or parish nursing, is too manifest to need further comment. One point, perhaps, needs explanation; the term "Regular Probationer" simply means that the probationer in question is bound for two years and receives payment from the hospital, and by no means implies any social distinction.

**Paying Probationers.** In addition to these regular probationers, in November, 1882, "Paying Probationers" were accepted for the first time, *i.e.*, ladies *and others* who were desirous of entering for short periods of training. From that time to the present, 246 paying probationers have been received. This number is exclusive of the frequent renewals of the "three months' engagement" by those who have once entered for that period. The terms are thirteen guineas, payable on entrance, but no certificate or testimonial is granted under the full term of two years' training. The only distinctions made between paying and regular probationers are, that the former are not bound for any specified time, they are exempt from night duty except at their own special request, and the limitations with respect to age which affect the selection of the regular staff are not adhered to in the case of paying probationers. The social position of paying and regular probationers is immaterial as far as their hospital career is concerned, or at any rate as far as it affects the period of training. The work is exactly the same for probationers of every class and upon whatever terms they enter.

**The Advantages of a large Hospital.** Many people who are ignorant of the details of hospital routine, labour under the impression that the work of such a large hospital must necessarily be extremely heavy. They overlook the fact that the staff is large in proportion to the claims upon them, whereas, in small hospitals, it frequently happens that the accommodation is too limited to admit of extra hands being retained; and, in the emergencies which must of necessity arise in institutions of this kind, much pressure inevitably falls to the share of the few available workers.

**Further Modifications desirable.** I am of opinion that something remains to be done in the way of lessening the amount of housework which nurses are, almos universally, called upon to perform. As matters stand at present, it is satisfactory for us to know that all members of our nursing staff are exempt from everything that could be strictly characterised as *heavy* work, and *scrubbing* of every kind is entirely taken off them. This is scarcely the occasion on which to discuss the respective advantages and disadvantages of admitting probationers for short periods of training into our large training schools. There is a good deal to be said on both sides of the question, both from the hospital point of view and from that of those members of the public who are so eager to avail themselves of the opportunities offered. It may be as well to state that our experience of this arrangement is in favour of its adoption at all *large* hospitals, *provided no petty distinctions are made* between those who are there to suit their own convenience, and those who have entered solely for the purpose of earning their livelihood. The system of making minor distinctions between "lady nurses" and others, appears to us too fallacious to need further comment. It can scarcely be otherwise than an advantage for any woman to be what we designate a "lady"; but this fact will not make her a trained nurse, unless she has qualified herself in a *thorough* manner.

**Half-Timers.** In addition to regular and paying probationers, the London Hospital Training School receives about a dozen nurses at one time, who are sent from various private nursing institutions, for half the fees required from the paying probationers. These enter for periods of not less than six months, and no certificate is accorded; but doubtless a term of six months in our busy wards gives them wider experience than a year in a quiet provincial hospital might do.

### A DISTRICT NURSE AND HER PATIENTS.

We have received the following contribution for the East London District Matron, whose article was published in The Hospital of 18th December:—"A Lancashire Lass," three shillings.

